# Honey DNA metabarcoding revealed foraging resource partitioning between Korean native and introduced honey bees (Hymenoptera: Apidae)

**DOI:** 10.1038/s41598-022-18465-5

**Published:** 2022-08-23

**Authors:** Saeed Mohamadzade Namin, Min-Jung Kim, Minwoong Son, Chuleui Jung

**Affiliations:** 1 Agricultural Science and Technology Institute, Andong National University, Andong, Republic of Korea; 2 Department of Plant Protection, Faculty of Agriculture, Varamin-Pishva Branch, Islamic Azad University, Varamin, Iran; 3 Department of Plant Medicals, Andong National University, Andong, Republic of Korea

**Keywords:** Ecology, Agroecology

## Abstract

Honey DNA metabarcoding provides information of floral sources of honey and foraging plant preferences of honey bees. We evaluated the floral composition of honey from two different species of honey bees, *Apis cerana* honey (ACH) and *A. mellifera* honey (AMH) in a mixed apiary located in a semi-forest environment to understand the floral preference and level of interspecific competition on floral resource. Three honey samples were collected from different hives of each species in mid-August. In total, 56 plant taxa were identified across the honey samples and among them, 38 taxonomic units were found in ACH compared with a total of 33 in AMH. The number of major plants (> 1% of reads) in honey samples was 9 and 11 in ACH and AMH respectively indicating the higher diversity of plant taxa in AMH. 23 taxonomic units were found exclusively in ACH, 18 taxonomic units were found only in AMH and 15 taxonomic units were shared between ACH and AMH indicating that 73% of the taxonomic units were present only in honey originated from one of the honeybee species. Qualitative and quantitative analyses of the shared major plants revealed the division of floral resource between these co-existing honey bee species pointing to a low level of interspecific competition between these two important pollinators.

## Introduction

Among all insects, honey bees are considered to be one of the most important pollinators^1^. Animal pollination directly affects about three-quarters of essential crop types, including most fruits, seeds, and nuts^2^. Two species of honey bee exist in South Korea: *Apis cerana* (the Eastern honey bee), which is a native honey bee and *A. mellifera* (the European honey bee), which is an exotic bee introduced to Korea in the early 1900s. The native bees are usually kept in logs or smaller boxes with movable frames, while the European honey bees are mostly housed in the standard Langstroth hives^3^. Because of the greater honey production of *A. mellifera*, commercial interest in the European honey bee has risen in the last decades^4^ and resulted in a gradual decline of *A. cerana* colonies in Japan and South Korea^3^. On the other hand, *A. cerana* is more suitably adapted to East Asian environmental conditions with long periods of rainfall^5,6^ as well as being more resistant to parasitic mites^7^, native predators^8^ and local diseases^9^.

Previous studies on the foraging behavior of these honey bees showed that *A. cerana* is able to collect more nectar in resource-scarce areas. In addition, their ability to start foraging at lower temperatures makes them suitable to pollinate flowering plants in late winter and early spring. In summer, *A. cerana* workers start foraging earlier than *A. mellifera* in the morning and stop foraging later in the evening resulting in approximately one extra hour of foraging than *A. mellifera*^5,10^. However, there are some foraging traits that would allow *A. mellifera* to outperform *A. cerana*. *A. mellifera* performs better than *A. cerana* during an odor acquisition phase^11^. *A. mellifera* has a significantly longer maximum foraging distance (over 10 km)^12^, compared to *A. cerana*’s 1.4–2.5 km^13,14^. In an apple orchard and broccoli field, *A. cerana* visited a higher number of flowers than *A. mellifera* per minute due to the longer times that *A. mellifera* remains on each flower than the native *A. cerana*^15^. A contradictory study found that there was no significant difference between the two species for number of flowers visited per minute, however, it is generally believed that *A. mellifera* visits a higher number of flowers on each foraging trip since their foraging trips cover a greater distance^10^ and they are able to carry heavier pollen loads^10,15^.

Since the introduction of exotic pollinators can lead to negative consequences on survival, growth, reproduction, and foraging behavior of native pollinators^16–19^, resulting in declines and extinctions of native plants, possessing information on the effect of the introduction of *A. mellifera* as an introduced honey bee on foraging activities of local pollinators is of crucial importance. Considering the ability of honey bees to fly hundreds of meters, monitoring the foraging behavior and floral preference of several bees simultaneously is challenging over a large area^20^.

The presence of pollen grains inside honey not only indicates the floral composition of honey, but also reflects the foraging behavior of honey bees. Furthermore, it gives long-term information about foraging activities of honey bees throughout the season. The traditional method of recognition of floral composition of honey is based on melissopalynology, which is an identification of pollen grains inside honey using light microscopy^21^. This method is tedious and time-consuming requiring expert palynologist in different groups of plants. If the pollen grains from phylogenetically close species of plants are similar morphologically, the taxonomic resolution of plant identifications through this method to species or at least genus level is often impossible^22,23^. DNA barcoding allows for identification and classification of organisms based on a short nucleotide sequence. Mixed origin, environmental samples, such as pollen, are characterized by the presence of DNA from different organisms. Next‐generation sequencing (NGS) technologies now allow high‐throughput sequencing of complex DNA libraries including pollen^24–26^. DNA metabarcoding, which is combination of NGS and DNA barcoding, can be applied in identifications of mixed origin pollen loads and surpass in reliability the traditional microscopic identification methods employed by palynology or mellisopalynology by detecting a greater number of taxa and tracing infrequently detected species^23,24^.

Ideal DNA barcodes have significant interspecific genetic variation and are flanked by conserved regions for universal primer binding to allow easy amplification across a wide range of taxa^27^. There is still a debate on the optimal DNA barcode for plants, and different molecular marker candidates were used for plant recognition through DNA barcoding such as chloroplast ribulose-bisphosphate carboxylase (rbcL), maturase K (matK), tRNA gene (trnL), intergenic psbA-trnH spacer and ribosomal internal transcribed spacer 2 (ITS2)^28,29^. All of them were used in the recent pollen DNA metabarcoding studies by Keller et al.^24^, Richardson et al.^25^, Sickle et al.^26^. Among them the rbcL chloroplast gene has been suggested as reliable candidate genes for plant identification which its highly universal primers are available^30^. Furthermore, recent detailed studies indicated the reliability of quantitative analyses based on plastid markers such as rbcL, trnL and matK^24,31–33^.

Since research on comparisons of floral preference in different species of honey bees is limited and most of the information is based on visual records in the field, the aim of this research has been to study floral composition of honey from two different honey bees, namely *A. cerana* and *A. mellifera* using the rbcL metabarcoding approach. In addition, possessing some knowledge on foraging preferences could assist us in long-term conservation programs aimed at rare and endangered plant species which these honey bees are prone to collect pollen and nectar from them.

## Results

### Sequencing output

**Figure 1:**
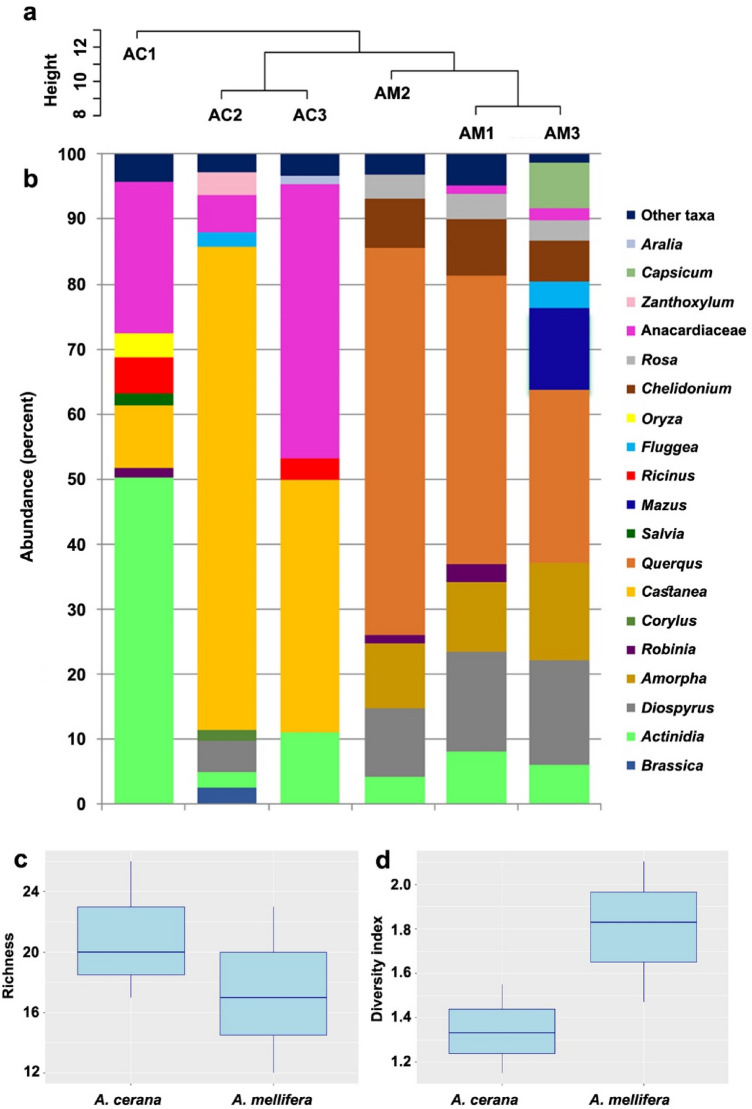
(**a**) The Euclidean distance–based dendrogram (top) summarizes the differentiation among honey samples from *Apis melifera* (AM-n) and *Apis cerena* (AC-n). (**b**) Bar charts showing the taxonomic composition at genus level in the six honey samples. Abundances of taxa are reported with the percentage values of reads. Taxa accounting for < 1% of reads are grouped as “Other taxa”. (**c**) Boxplots of the taxa richness in honey samples from *A. cerana* and *A. mellifera*. (**d**) Boxplots of Shannon diversity index of *A. cerana* honey and *A. mellifera* honey. P values are based on Mann–Whitney U test.

The total sample consisted of three ACH and three AMH and one negative control. In total 477,674 sequences obtained from six honey samples and initial quality control based on quality score, adaptor and length trimming resulted in 377,391 high-quality merged reads (Supplementary table 2). Sequences were compared against the NCBI database, with 99.4% being characterized to family, genus or species level. In total, 57 plant taxa were identified across the honey samples. Most (64.3%) were identified to genus level, 30.1% were identified to species and the remaining 5.6% to family level. Although significant numbers of the taxonomic units were recognizable to species level, there is a possibility to have false positives as reference sequences of several species from related genera are lacking, so that the data related to genus level alone are presented in this study (Table 1, Fig. 1).

**Table 1:**
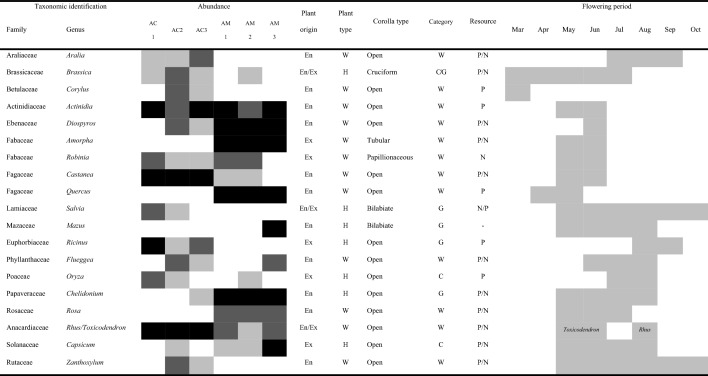
Plant taxa with > 1% abundance in at least one of the honey samples, and the status of the recorded plants.

The presence of plants within the honey sample is shaded in gray for each hive. Supplementary table 3 provides the proportions per hive and total in percent. The status of each genus is a consensus information collected from Supplementary table 4 which is based of all species of each genus available within Korea. Abundance: Light gray: < 1%, dark gray: > 1% and < 5%, black: > 5%. Plant origin: whether the plant genus contain species with endemic origin En or exotic origin Ex. Plant type: woody tree, shrub and vine W, herbaceous H. Corolla type data is based on information which is available in Bosch et al.^68^, Endress^69^, Gómez et al.^70^, and Watts et al.^71^. Category: Woodland W, grassland G, crop C. Resource: whether honey bees use the plant for nectar N or pollen P according to Sasaki^51^ and Simpson^52^. Flowering period: Consensus period of flowering of all species of related genus in Korea (available in Supplementary table 4) according to Lee^72^.

### Floral composition of honey of *Apis cerana* and *A. mellifera*

**Figure 2:**
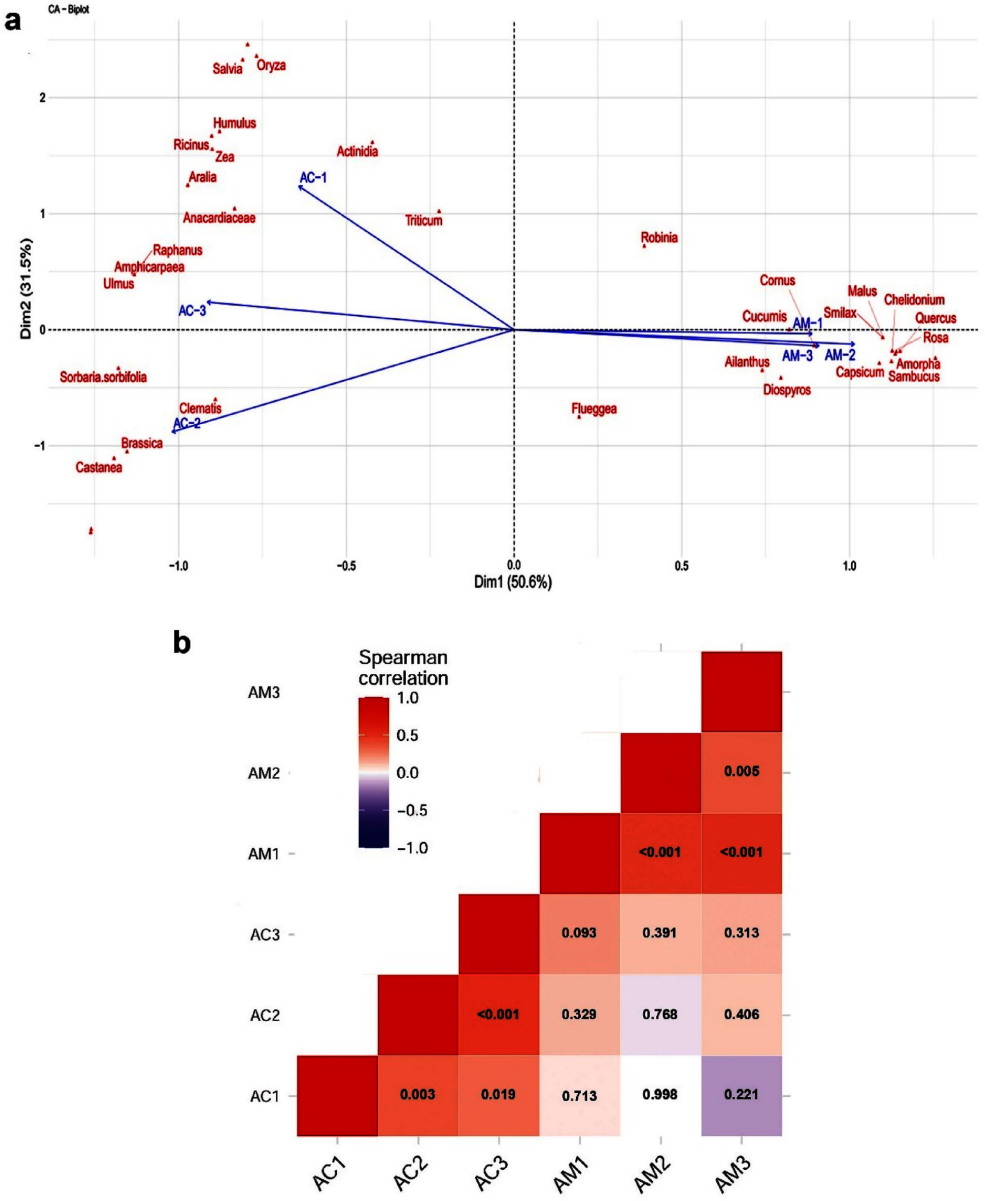
(**a**) Correspondence analysis (CA) representing the plant taxa and their abundances in the honey samples from *Apis melifera* (AM-n) and *Apis cerena* (AC-n). (**b**) A heat map representation of the Spearman correlation matrix of the six honey samples based on floral composition. The numbers indicate the p-value of the correlation.

In total, 23 taxonomic units were found exclusively in ACH; 18 plant taxa were found only in AMH and 15 taxonomic units were shared between ACH and AMH. Although the number of taxonomic units was ranging from 17 to 26 (n = 3) in ACH samples and 12 to 23 in AMH, the difference in taxon richness between ACH and AMH was not significant (Mann–Whitney U test, W = 6.5, p = 0.506, N = 3) (Fig. 1c). In total, 38 taxonomic units were found in ACH in comparison to a total of 33 found in AMH. The Shannon diversity index ranged between 1.47 and 2.1 in AMH and 1.15–1.55 in ACH, but the difference between them was not significant (Mann–Whitney U test, W = 1, p = 0.190) (Figs. 1d and 2b).

At family level, a total of 37 plant families (S1 Dataset) was determined with 14 families shared between ACH and AMH; 14 families were found only in ACH and 8 families in AMH. We examined the proportion of the taxonomic units of the honey samples and in ACH, 9 (23.1%) major taxa (> 1% of reads) were detected whereas the number of major taxa in AMH was 11 (32.3%) (Supplementary table 3).

**Figure 3:**
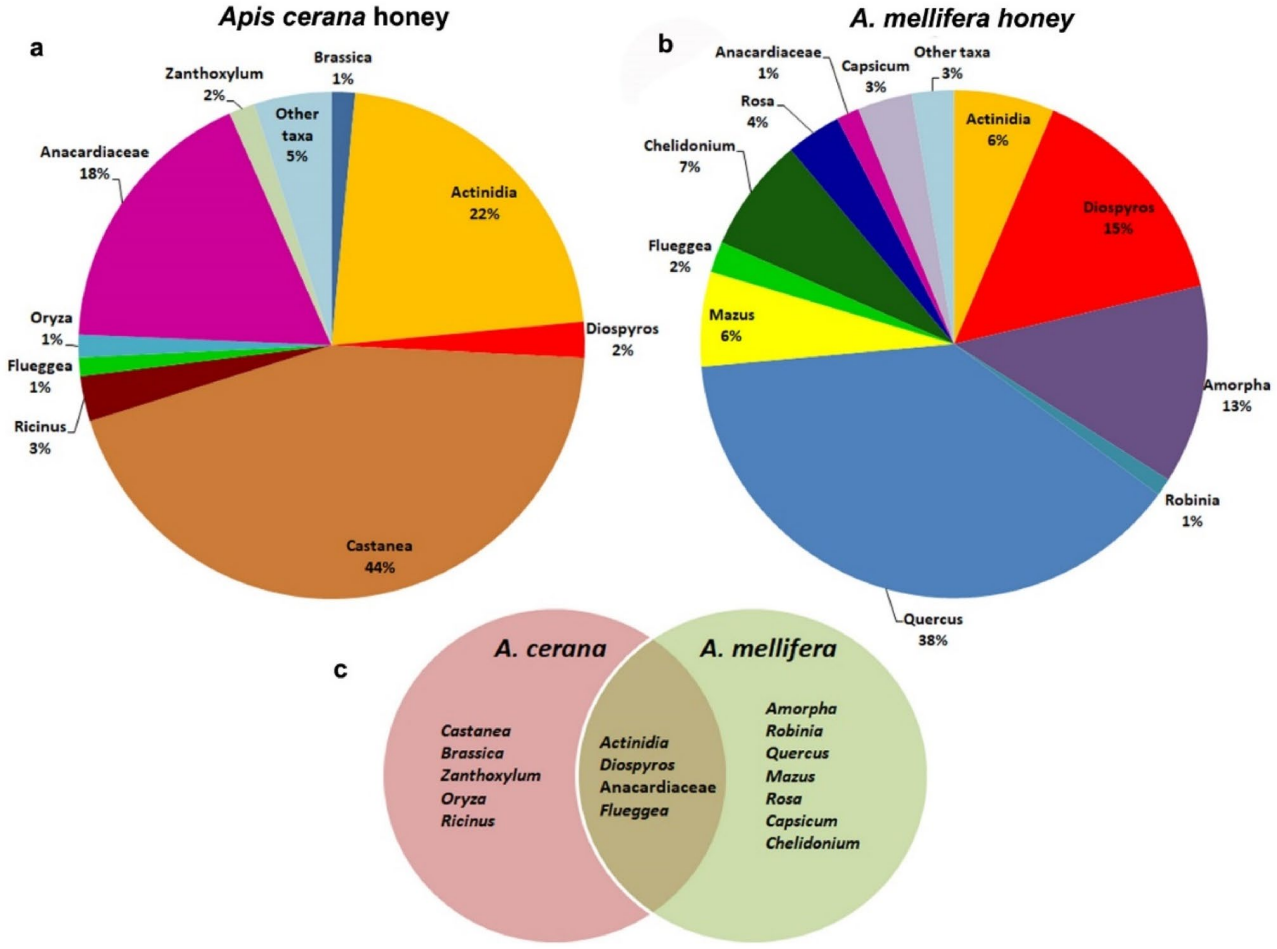
(**a**, **b**) Pie charts showing the taxonomic composition of major plants (> 1%) at genus level in *A. cerana* honey and *A. mellifera* honey. Abundances of taxa are reported with the percentage values of reads. Taxa accounting for < 1% of reads are grouped as “Other taxa”. (**c**) Venn diagram shows the number of unique and shared taxa identified at the genus level among honey samples.

*Castanea* were found in a higher proportion in ACH including 44 percent of the reads, following by *Actinidia* and Anacardiaceae (*Rhus*/*Toxicodendron*) with relative abundance of 22% and 18%, respectively. Other major plant genera were *Ricinus*, *Diospyros*, *Zanthoxylum*, *Brassica*, *Oryza* and *Flueggea* found in a relatively high proportion (with relative abundance of 1.2–2.9%) (Table 1, Fig. 3). The results showed that in ACH, 95% of reads were represented by these taxonomic units and the remaining 29 taxonomic units comprised only 5% of the reads (Supplementary table 3). In AMH, *Quercus* is the dominant taxonomic unit with relative abundance of 38.5% of total reads followed by *Diospyros*, *Amorpha*, *Chelidonium*, *Actinidia*, *Mazus*, which are the other common taxonomic units with relative abundances of 14.9%, 12.7%, 7.3, 6.4%, 6% respectively, followed by *Capsicum*, *Rosa* and *Flueggea* and *Robinia* with relative abundances of 1.1–3.5% (Table 1, Fig. 3). In AMH, 97.3% of the reads represented 11 major plant taxa and the remaining 22 taxonomic units comprised only 2.7% of the reads (Supplementary table 3).

The intraspecific pairwise comparison of honey samples showed that there was a significant correlation in abundance of plant taxonomic units in ACH and AMH (p < 0.025 for all pairwise comparison) with Spearman’s Rho ranging from 0.309 to 0.544 in ACH and from 0.363 to 0.521 in AMH. The interspecific pairwise comparison of ACH and AMH was not significant (p > 0.025 for all pairwise comparison). The correspondence analysis (CA) result clearly showed the major plant species that the two different honey bee species visit, were different from each other (Fig. 2a). AMH showed relatively less intra-colony variability than ACH samples in floral compositions.

*Actinidia* and *Rhus*/*Toxicodendron* are the only taxonomic units found in all samples. Besides these taxonomic units, *Castanea*, *Brassica* and *Ricinus* are the major plants and *Aralia*, *Robinia* and *Zea* are the minor taxonomic units which are available in all ACH samples, whereas *Quercus*, *Driospiros*, *Amorpha*, *Chelidonium* and *Capsicum* are taxonomic units (all are among major taxa), which are present in all AMH samples (Table 1, Fig. 1b, Supplementary table 4).

## Discussion

In this study, two species of honey bees, *A. cerana* and *A. mellifera*, had access to a wide range of agricultural and wild flowering plants in a semi-forested region. The study aimed to identify and understand the foraging preferences of these two important pollinators in a mixed apiary. In total 56 taxonomic units (genus level) were detected in the honey samples. According to the results, the number of taxonomic units which are exclusively presented in ACH and AMH was 23 and 15, respectively, showing that 73% of the taxonomic units were present only in one type of honey. In addition, both ACH and AMH shared *Actinidia*, *Diospyros*, *Flueggea*, and Anacardiaceae in the list of their major plants (Fig. 3c) which all together covered 43% and 25% of the total reads of ACH and AMH, respectively. Among them, *Actinidia* and Anacardiaceae included 40% of the total reads in ACH while representing only 7% of the reads in AMH. On the other hand, unlike AMH with *Diospyros* included to 15% of the total reads, it is only covered to about 2% of the reads in ACH. These differences clearly reveal the partitioning of foraging floral resources between *A. cerana* and *A. mellifera* (Table 1, Supplementary Fig. 3).

Pollen is a source of lipid, protein and micronutrients, necessary for growth and health of honey bees^34^ and its quality as well as other factors such as availability of plants within the landscape and quantity of nectar and morphology of flowers are crucial factors for bees to collect pollen from^35,36^. In addition, Pollen grains attach to honeybee body while visiting flowers during nectar collecting and study the pollen grains inside honey indicates the plants visited by honeybees^37^. One possible reason for the presence of different major plants in the two types of honey is the difference in nutritional requirements of these two species for healthy growth of their colonies; however, the absence of *Castanea* which is an important foraging target for *A. mellifera* among major plants of AMH is quite surprising. Another possible reason for visiting different flower resources by *A. cerana* and *A. mellifera* is due to the avoidance of interspecific competition. Previous studies have shown that the introduction of *A. mellifera* can reduce survival, growth, reproduction, and have a negative impact on feeding behavior of native pollinators^17,18^. Since *A. cerana* is a close relative of *A. mellifera*^38^, the introduction of *A. mellifera* in *A. cerana* distribution areas can cause devastating ecological effects such as cross-species mating with *A. cerana* and reductions of the native species’ fitness^39^, exchange of pests and diseases and more importantly interspecific competition on floral resource^19,40^. These negative effects have caused regional extinctions of the *A. cerana* in Japan^41^, India^16^ and China^19^. The negative effects of introducing an exotic pollinator may lead to behavioral modifications of endemic ones such as timing of flower visitations to avoid competition with introduced honey bees^42^. The introduction of the European honey bee into *A. cerana*’s territory had considerable negative effects on *A. cerana*’s population and the plants used to be pollinated by *A. cerana*. However, due to resource partitioning between the introduced and native honey bees, after about one hundred and twenty years of its first introduction, *A. mellifera* like *A. cerana* can both be considered to be important pollinators for Korean crops and wild flowering plants, showing that the preservation of both species must be taken seriously. Loss of a single pollinator can cause significant implications for plant communities due to the alteration in floral fidelity in the remaining pollinators and a reduction of plant reproduction^43^. Iwasaki et al.^44^ showed the resource partitioning between introduced bees, *A. mellifera* and *Bombus terrestris*, with New Zeeland native solitary bees indicated limited resource overlap between bee taxa being the result of low competition due to different nutritional requirements and the pollinators’ abilities to access floral resources. The presence of both honey bee species in the same apiary can lead honey bees to modify aspects of their behavioral repertoire and to avoid competition for floral resources and to provide pollination services for higher numbers of flowering plants in nature. Most previous research focused on the pollination network among a coexisting pollinator community^44–46^. However, there is still only limited information available about the impact of resource partitioning on colony growth and survival of pollinators which is worthwhile to be taken into consideration in future studies.

The richness of the taxonomic units in the ACH was slightly higher than that of the AMH, resulting in a higher taxa diversity index of AMH indicating that the proportion of major plants was higher in AMH. However, the differences in taxa richness and diversity index between ACH and ADH were not significant which it is due to the low sample size. Lucek et al.^47^ also mentioned the requirement of high sample size to detect a statistical difference in taxa richness and diversity index between honey samples collected from urban and non-urban sites. Previous studies also demonstrated that *A. cerana* visited more flowering plants than *A. mellifera*^48^ and exhibited a higher performance while collecting pollen from scattered flowers of a variety of plant species, making more foraging trips per day and spending less time on each flower^13,15,49^. Being a native species of Asia, *A. cerana* commence foraging earlier in the day than *A. mellifera* and initiate foraging earlier in spring due to their ability to tolerate lower temperature (Supplementary Fig. 2). *A. cerana* also have access to a wider range of flowering plants to collect nectar and pollen from and the presence of *Corylus* among major plants of one of the *A. cerana* honey samples is indicative of the foraging activities of the recent species in early March (Table 1). Another plant taxon providing early season flowers is *Brassica* presented among major plants and identified from all ACH samples.

The list of taxonomic units of ACH and AMH contains several major plants followed by a long tail of minor plants. Previous melissopalynological or DNA metabarcoding studies on floral composition of honey also indicated that honey bees use a wide range of flowering plants with a relatively limited number of core plants^22,36,50^. Although the most abundant plants in the studied area were coniferous plants covering about 40% of landscape and about 60% of vegetation covered area (Supplementary table 1), *Cerdus* was the only taxonomic unit presented among minor plants which was visited exclusively by *A. mellifera* (Supplementary table 3).

The majority (92% of reads) of major plant taxa from ACH were the woody trees and shrubs including *Castanea*, *Actinidia*, *Diospyros*. *Ricinus*, *Flueggea*, Anacardiaceae, and *Zanthoxylum*. Likewise, in DNA extracts from AMH, apart from *Capsicum*, *Mazus*, and *Chelidonium*, the rest of the major plants (80% of total reads) were among trees and shrubs which included *Quercus*, *Actinidia*, *Diospyros*, *Amorpha*, *Flueggea*, Anacardiaceae and *Rosa*. Among horticultural plants, *Brassica* and *Oryza* were the major plants in ACH while *Caspicum* was present in AMH (Table 1, Supplementary table 3).

Both *A. cerana* and *A. mellifera* preferred to collect pollen from open type of corolla; the corolla type is quite diverse among major plants recognized from the honey produced by both species, however, *Amorpha* which have a tubular type of corolla found only in AMH. Most of the major taxa collected in this study by honey bees were among pollen-rich flowering plants except *Robinia,* which is an excellent source of nectar^51,52^. Moreover, *Querqus* and *Ricinus* which rarely become targets for honey bees as foraging plants, were among the major plants of *A. mellifera* and *A. cerana,* respectively. Previous studies on the foraging behavior of two species of stingless bees (*Melipona bicolor* and *M. quadrifasciata*) and *A. mellifera* showed that niche overlaps were more evident for nectar than for pollen^53^. *Robinia*, *Amorpha* and *caspicus* are introduced taxa, originating from Central and North America (Supplementary table 4), found as major plants of AMH, including 17% of the total reads whereas in ACH, *Robinia*, *Ricinus* and *Oryza* are the introduced major taxa with only about 5% of reads. *Ricinus* originated from Africa while *Oryza* was introduced from China (Supplementary table 4) where *A. cerana* is a native species^14^. En/Ex (Table 1) attributed to the major taxa containing at least one introduced and one endemic species in Korea and among them Anacardiaceae is one of the most abundant taxa in ACH with relative abundance of 17.7% while only covering 1.5% in AMH. The only introduced plant in Anacardiaceae, *T. vernicifluum*, is native to India and China (Supplementary table 4) where *A. cerana* is also endemic^14^. In total, compared with ACH, the abundance of introduced plant taxa in AMH is slightly higher than in ACH, which is congruent with previous studies in Japan where *A. cerana* visited native plant species more often than *A. mellofera*^48^. Nevertheless, some of the endemic taxa such as *Quercus*, *Mazus*, and *Rosa* are exclusively found in AMH.

Honey bees collect nectar and pollen from visiting flowers during their foraging trips to provide nutrients required for their colony development^54^. Nectar is processed to form honey. However, some pollen grains are also available in the honey allowing us to analyze the preferences that the bees have for certain species of foraging plants. In this study, the DNA was extracted from honey pellets after centrifuging. One possible reason for the fact that DNA metabarcoding is able to detect hidden and low abundant taxa in comparison with mellisopalynology^22^ is that the DNA can be also extracted from the plant cells which are available in the nectar through DNA metabarcoding. Recent studies indicated the presence of different groups of organisms such as Viruses, Bacteria, Arthropoda, Fungi, Plants and even vertebrates in the DNA extracted from pellets of the centrifuged honey^55^. Prosser and Hebert^56^ also documented the presence of different plant taxa inside DNA extract from the liquid part of the honey and the pellet as well. However, since different markers were used for liquid and pellet we were not able to discuss their taxon richness and diversity and it has to remain a task for future studies.

## Methods

### Honey samples

Three *A. cerana cerana* and three *A. mellifera ligustica* hives with healthy queens from a mixed stationary apiary, were selected randomly for sampling. *A. mellifera* colonies were kept in 10-framed langthroth hives while *A. cerana* colonies were kept in smaller size modern hives with 8 movable frames designed for Eastern honeybee. The apiary containing 10 *A. cerana* and 30 *A. mellifera* and located in a semi-forested hilly area with some agricultural fields of rice, potato, tomato, and pepper and small orchards of jujube, peach and cherry nearby, on the western coast of Andongho Lake, Andong, South Korea (reservoir, 36°40′00″ N, 128°49′34″ E, 177 m) (Supplementary Fig. 1). Five new frames were introduced into each colony of both honeybees in early March and Honey samples were harvested from introduced frames on the 15th August 2020 and 500 g honey from each blended sample were stored for forthcoming analyses.

Landscape analysis was conducted over a 2.5 km radius around the apiary to identify landcover types. The radius was decided based on foraging distances of both *A. mellifera* and *A. cerana*^12,13,57^. We used landcover map data with 1 m spatial resolution from the Korea Ministry of Environment (KME) website (http://eng.me.go.kr/). A circle of 2.5 km radius was given to the base map and the landcover types were extracted in ArcGIS pro^58^. In the given circle, potential foraging habitats for nectar of 14.3 km^2^ were identified. Among the forest types, coniferous, broad-leaf and mixed forests were dominant, followed by agricultural fields and grassland (Supplementary table 1).

### DNA extraction

Total DNA was extracted using a modified version of protocol of DNeasy Plant Mini Kit (Qiagen) for DNA isolation from honey used by de Vere et al.^36^ with modification on preparing samples. Briefly, 10 g of each honey were used for DNA extraction. DNA from each of the honey samples extracted four times and mixed together before library preparation. The honey sample was placed into a sterile 50 ml centrifuge tube and 30 ml pure water was added. Samples were incubated at 65 °C for 30 min with shaking in every five minutes, and then centrifuged for 30 min at 15,000 g. The supernatant was discarded and each pellet was resuspended in 10 ml ultrapure water and after homogenizing centrifuged again for 30 min at 15,000 g. The pellet resuspended in 1 ml water and transferred to 1.5 ml eppendorf tube and centrifuged for 10 min at 15000 g and the sediment were used for DNA isolation following the protocol by de Vere et al.^36^. An isolation negative control was also included using 40 ml of ultrapure water. The extracted DNA stored at − 20 °C prior to library preparation.

### Library preparation and sequencing

Two rounds of PCR were applied for library preparation of rbcL DNA barcode marker region. The first PCR was conducted to amplify the rbcL region using the universal primers rbcL2f^60^ and rbcLaR^61^ to which adaptor ‘tails’ had been added and the second PCR was applied to attach unique tags for separation of the sequences into samples after sequencing. PCR was conducted in a final volume of 25 μl. A total of 2.5 μl of template DNA was combined with 12.5 μl of 2 × KAPA HiFi HotStart ReadyMix (KAPABIOSYSTEMS) and 5 μl of each primer (1 μM). The reaction was performed following program: initial denaturing at 95 °C for 3 min, followed by 30 cycles of 95 °C for 30 s, 50 °C for 30 s, 72 °C for 30 s with a final extension at 72 °C for 5 min. Products from the first PCR were purified using AMPureXP purification kit. The second PCR was conducted in a final volume of 50 μl using 5 μl of the first PCR product as a template, mixed with 25 μl of KAPA HiFi HotStart ReadyMix, 5 μl of Nextra XT Index Primer 1 (N7xx), 5 μl of Nextra XT Index Primer 2 (S5xx) and 10 μl of PCR Grade water. The PCR reaction was repeated as for the first PCR but with 8 cycles. The resulting products were purified using a AMPureXP purification kit. The pooled mixture was sequenced in a single flow cell on a 600-cycle run of the Illumina MiSeq instrument (Illumina, USA) at Macrogen (South Korea). All sequence information has been deposited in the National Center for Biotechnology Information (NCBI) Sequence Read Archive (accession code PRJNA768940).

### Bioinformatic analysis

Raw FASTQ files were demultiplexed by the sequencing company. Reads were trimmed for presence of adapter sequence using Trimmomatic^62^. Paired-end reads were quality-controlled considering the phred scale quality score trimming threshold, which was set to 25 and only the paired-end reads more than 180 bp after trimming were kept. Forward and reverse reads were merged using VSEARCH 2.14.1^63^ and the merged reads lesser than 450 bp and quality score lower than 20 were discarded using SICKLE version 1.33^64^. Length trimmed sequences were converted to FASTA format and dereplicated using VSEARCH. An rbcL reference database was assembled as described below using OBITools3^65^. All available relevant sequences were downloaded from EMBL database (2021.04.17), dereplicated and trimmed based on same primer sets used in this study and a formatted taxon list was created using only taxa with complete binomial species names. The assembled reference database was converted to FASTA format and used to check potential chimeras using uchime-denovo command in VSEARCH. Chimera free sequences were clustered at 97% threshold using VSEARCH. The BLASTN algorithm (blast-2.11.0)^66^ was used to align representative sequences with the GenBank derived sequence library (20201.06.21) using the following settings: E-value cutoff 1e−125, number of alignments 5, output format 0, number of descriptions 10, percent identity threshold 95%. The species name assigned to the sequence when the top bit score is matched to a single species. If the top bit score be the same for different species belonging to the same genus, then the sequence identified to that genus. A family level assigned to the sequence when the top bit score matched to multiple genera within the same family. The sequences not identified in family level were discarded. The scientific name of OTUs matched with the list of Korean plants (http://koreanplant.info/wordpress/index.php/plantlist-eng/) and the ones with species name not available in the list recognized in genus level. To reduce sequencing errors, OTUs with lesser than 10 reads were discarded. In addition, the negative control was used to set threshold values for sequence removals and any taxonomic classifications recorded from fewer reads than the read number we obtained from a negative control were removed from further analysis. The relative abundances of each taxon in percentage were used as a semi-quantitative measurement to demonstrate the honey bees’ foraging behavior.

### Statistical analysis

Statistical analyses involved R 4.1.0. Spearman’s Rank Correlations for multiple testing were used to assess whether each colony of honey bees used the same plants in similar proportions. Differences between taxa richness and Shannon diversity index from the samples of each group (*A. cerana* and *A. mellifera* honey samples) were compared using Mann–Whitney U test. Spearman’s Rank Correlations and Mann–Whitney U tests were used as the data did not meet the assumption of normality required for parametric tests.

We conducted correspondence analysis (CA) to visualize the relationships between plant taxa and their abundances in the honey samples from *A. melifera* and *A. cerena*. CA was conducted with “FactoMineR” package in R 4.1.0^67^. A hierarchical clustering analysis (HCA) was also conducted based on plant species information to identify whether combination of floral sources can specify foraging characteristics of two honey bee species, *A. melifera* and *A. cerena*. The ward algorithm with minimum variance method was used for the clustering with “cluster” package in R 4.1.0^67^. The clustering result was visualized in a dendrogram plot.

## Supplementary Information

Supplementary Information.

## Data Availability

All sequence information has been deposited in the National Center for Biotechnology Information (NCBI) Sequence Read Archive (accession code PRJNA768940).
