# Supplementary material for: Honey DNA metabarcoding revealed foraging resource partitioning between Korean native and introduced honey bees (Hymenoptera: Apidae)
Source: Sci Rep. 2022 Aug 23;12:14394. doi: 10.1038/s41598-022-18465-5 (PMC9399230; doi:10.1038/s41598-022-18465-5)
Supplement: Supplementary file 1 — Supplementary Information. [file 41598_2022_18465_MOESM1_ESM.pdf]

Honey DNA metabarcoding revealed foraging resource partitioning between Korean native and introduced honeybees (Hymenoptera: Apidae)

Saeed Mohamadzade Namin, Min-Jung Kim, Minwoong Son, Chuleui Jung

Supplementary table 1. Landcover composition of 2.5 km radius around apiary where honey samples were collected.

Habitat	Type	Attribute	Proportional area (%)
**(Semi-) natural plant habitat**	Natural	Grass land	3.71%
		Coniferous forests	42.18%
		Mixed forest	5.49%
		broadleaf forest	11.87%
	Agricultural	Rice	3.55%
		Crop	5.47%
		Orchard	0.35%
**Non-plant habitat**	Natural	River	3.04%
		Lake	16.22%
		Rock	5.23%
	Agricultural	Greenhouse	0.04%
		Farm and fishery	0.03%
	Disturbed	Facility	0.03%
		Residential area	0.25%
		Road	1.06%
		Graveyard	1.15%
		Bare land	0.34%

Supplementary table 2. The number of raw reads per honey samples from *Apis melifera* (AM-n) and *Apis cerena* (AC-n) and the remaining reads after quality trimming, merging, length trimming, and chimera checking.

Type of honey	Sample ID	Number of reads	No of reads after quality trimming	NO. of merged reads	NO. of reads after length trimming	NO. of reads after chimera checking	NO. of reads assigned taxonomy
**A. cerana**	**AC1**	83992	78751	74692	74691	74629	74629
	**AC2**	104923	97401	93009	93006	91854	91806
	**AC3**	32402	29948	28253	28252	28220	27324
**A. mellifera**	**AM1**	98020	77738	61423	61423	61398	61324
	**AM2**	36662	34118	32190	32189	32111	32104
	**AM3**	121675	105806	89197	89195	89179	87974
**Negative control**	**NC**	855	623	485	479	479	375
**Total NO. of reads**		478529	424385	379249	379235	377870	375536

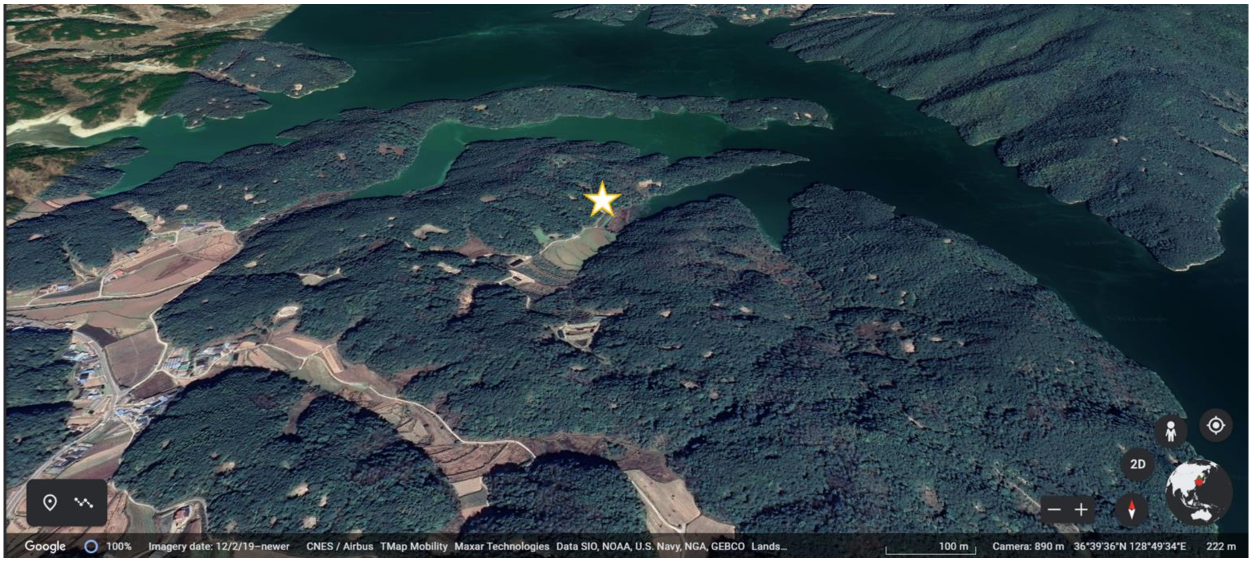

Supplementary figure 1. Map of the study area. Image adapted from Google Earth (https://earth.google.com/). The star indicates the location of the apiary.

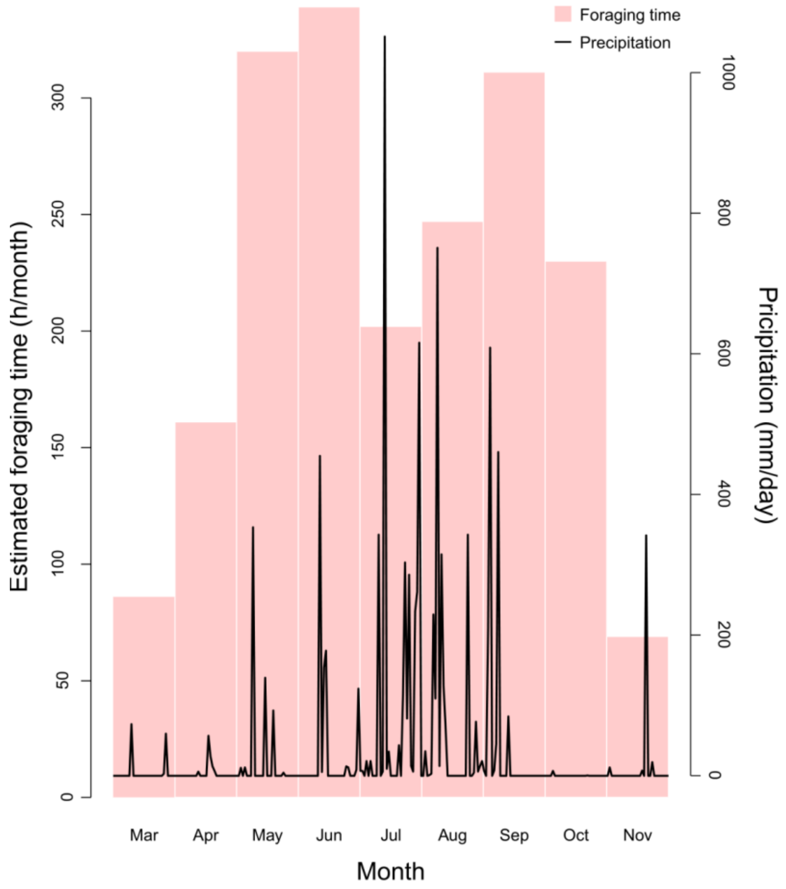

Supplementary figure 2. Precipitation (mm/day) in study area, and estimated foraging time (h/month) of honeybees. Foraging time was calculated as daytime (6AM – 19AM) when temperature was upper than 15 ºC without rainfall (Kim et al., 2021), using hourly weather data from Rural development Administration (RDA) website (http://weather.rda.go.kr).

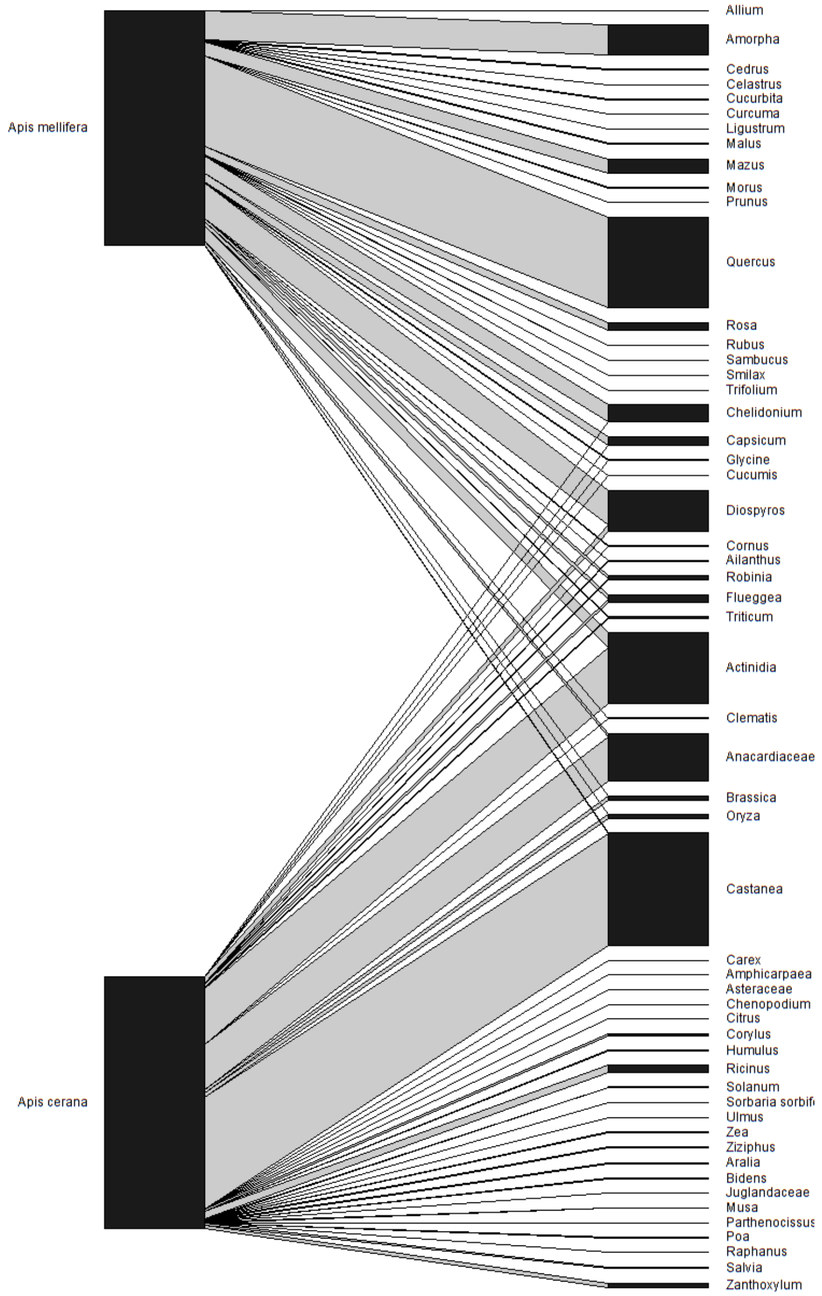

Supplementary figure 3. Bipartite graphs of the foraging plant preference of honeybees, *A. mellifera* and *A. cerana*. Right bars represent honeybee species and left bar represent plant taxa. Linkage width indicates the frequency of reads from each plant taxa in honey collected from a given honeybee species in this study.

Supplementary table 3. Taxonomic identification assigned to the sequences from six honey samples. The numbers in bold with tinge orange and blue background indicates the major plants (>1%) among honey samples from Apis melifera (AM-n) and *Apis cerena* (AC-n).

Taxonomic ID			AC1	AC2	AC3	AM1	AM2	AM3	Total AC	Total AM
Order	Family	Genus	Percentage	Percentage	Percentage	Percentage	Percentage	Percentage	Percentage	Percentage
Apiales	Araliaceae	Aralia	0.62	0.11	**1.17**	-	-	-	0.46	-
Asparagales	Amaryllidaceae	Allium	-	-	-	-	0.11	-	-	0.02
Asterales	Asteraceae	Erigeron/Conyza	-	0.11	-	-	-	-	0.05	-
Asterales	Asteraceae	Bidens	0.23	-	-	-	-	-	0.09	-
Brassicales	Brassicaceae	Brassica	0.51	**2.56**	0.65	-	0.15	-	**1.50**	0.03
Brassicales	Brassicaceae	Raphanus	-	-	0.04	-	-	-	0.01	-
Caryophyllales	Chenopodiaceae	Chenopodium	0.18	-	-	-	-	-	0.07	-
Celastrales	Celastraceae	Celastrus	-	-	-	-	0.13	-	-	0.02
Cornales	Cornaceae	Cornus	-	-	0.06	-	0.31	-	0.01	0.06
Cucurbitales	Cucurbitaceae	Cucumis	-	-	0.14	0.46	-	-	0.02	0.15
Cucurbitales	Cucurbitaceae	Cucurbita	-	-	-	-	0.49	-	-	0.09
Dipsacales	Adoxaceae	Sambucus	-	-	-	-	-	0.33	-	0.16
Ericales	Actinidiaceae	Actinidia	**50.26**	**2.37**	**11.13**	**8.13**	**4.18**	**6.00**	**22.03**	**6.40**
Ericales	Ebenaceae	Diospyros	-	**4.82**	0.05	**15.38**	**10.66**	**16.15**	**2.28**	**14.92**
Fabales	Fabaceae	Amorpha	-	-	-	**10.72**	**9.99**	**15.02**	-	**12.67**
Fabales	Fabaceae	Amphicarpaea	-	-	0.05	-	-	-	0.01	-
Fabales	Fabaceae	Glycine	-	-	-	-	-	0.84	-	0.41
Fabales	Fabaceae	Robinia	**1.47**	0.10	0.10	**2.70**	**1.15**	-	0.63	**1.12**
Fabales	Fabaceae	Glycine	-	0.04	-	-	-	-	0.02	-
Fabales	Fabaceae	Trifolium	-	-	-	-	0.30	-	-	0.05
Fagales	Betulaceae	Corylus	-	**1.74**	0.08	-	-	-	0.83	-
Fagales	Fagaceae	Castanea	**9.70**	**74.36**	**38.80**	0.70	0.29	-	**44.40**	0.29
Fagales	Fagaceae	Quercus	-	-	-	**44.41**	**59.49**	**26.71**	-	**38.50**
Fagales	Juglandaceae	Pterocarya/Juglans	0.01	-	-	-	-	-	0.01	-
Lamiales	Lamiaceae	Salvia	**1.80**	0.05	-	-	-	-	0.71	-
Lamiales	Mazaceae	Mazus	-	-	-	-	-	**12.43**	-	**6.02**
Lamiales	Oleaceae	Ligustrum	-	-	-	-	0.07	-	-	0.01
Liliales	Smilacaceae	Smilax	-	-	-	0.67	-	-	-	0.23
Malpighiales	Euphorbiaceae	Ricinus	**5.47**	0.68	**3.27**	-	-	-	**2.89**	-
Malpighiales	Phyllanthaceae	Flueggea	-	**2.23**	0.94	-	-	**4.06**	**1.19**	**1.97**
Poales	Cyperaceae	Carex	-	0.03	-	-	-	-	0.02	-
Poales	Poaceae	Zea	0.53	0.11	0.08	-	-	-	0.26	-
Poales	Poaceae	Oryza	**3.71**	0.04	-	-	0.14	-	**1.45**	0.03
Poales	Poaceae	Triticum	0.84	0.18	-	0.66	-	-	0.41	0.22
Poales	Poaceae	Poa	0.23	-	-	-	-	-	0.09	-
Ranunculales	Papaveraceae	Chelidonium	-	-	0.25	**8.56**	**7.68**	**6.29**	0.04	**7.30**
Ranunculales	Ranunculaceae	Clematis	-	0.22	0.69	0.03	-	0.05	0.20	0.03
Rosales	Cannabaceae	Humulus	0.33	0.06	-	-	-	-	0.15	-
Rosales	Moraceae	Morus	-	-	-	-	0.24	-	-	0.04

**Rosales**	**Rhamnaceae**	**Ziziphus**	-	0.66	-	-	-	-	0.31	-
**Rosales**	**Rosaceae**	**Malus**	-	-	-	0.95	-	-	-	0.32
**Rosales**	**Rosaceae**	**Prunus**	-	-	-	-	0.07	-	-	0.01
**Rosales**	**Rosaceae**	**Rosa**	-	-	-	**3.90**	**3.68**	**3.21**	-	**3.52**
**Rosales**	**Rosaceae**	**Sorbaria sorbifolia**	-	0.04	0.21	-	-	-	0.05	-
**Rosales**	**Rosaceae**	**Rubus**	-	-	-	-	0.15	-	-	0.03
**Rosales**	**Ulmaceae**	**Ulmus**	-	-	0.04	-	-	-	0.01	-
**Sapindales**	**Anacardiaceae**	**Rhus/Toxicodendron**	**23.33**	**5.73**	**42.21**	**1.27**	0.40	**1.94**	**17.75**	**1.44**
**Sapindales**	**Rutaceae**	**Zanthoxylum**	-	**3.50**	0.04	-	-	-	**1.66**	-
**Sapindales**	**Rutaceae**	**Citrus**	-	0.03	-	-	-	-	0.01	-
**Sapindales**	**Simaroubaceae**	**Ailanthus**	-	0.05	-	0.35	0.08	-	0.02	0.13
**Sapindales**	**Solanaceae**	**Capsicum**	-	0.10	-	0.31	0.04	**6.98**	0.05	**3.49**
**Sapindales**	**Solanaceae**	**Solanum**	0.62	-	-	-	-	-	0.24	-
**Vitales**	**Vitaceae**	**Parthenocissus**	-	0.06	-	-	-	-	0.03	-
**Zingiberales**	**Musaceae**	**Musa**	0.19	-	-	-	-	-	0.07	-
**Zingiberales**	**Zingiberaceae**	**Curcuma**	-	-	-	-	0.21	-	-	0.04
**Pinidae**	**Pinaceae**	**Cedrus**	-	-	-	0.81	-	-	-	0.28
**Total number of reads**			74631	91654	27987	61324	32102	87898	194,272	181,324
**Number of Major plants**			7	8	5	8	7	10	9	11
**Total number of plants**			17	26	20	17	23	13	39	34

Supplementary table 4. The species of the major taxa in at least one of the honey samples (genus or family with >1% abundance) which are available in Korea, and the status of the recorded plants. Plant origin: whether the plant genus contain species with native origin or exotic origin. Plant type: woody tree, shrub and vine W, herbaceous H. Category: Woodland W, grassland G, crop C. Corolla type data is based on information which is available in Bosch et al. (1997), Endress (2010), Gómez et al. (2016), and Watts et al. (2016). Color: color of flowers: Green G, Pink Pi, Purple Pu, Red R, White W, Yellow Y. Resource: whether honey bees use the plant for nectar or pollen (No information N, Rarely R, Incidentally I, Temporary T, Good G, Excellent E) according to Sasaki (2010) and Simpson (2010). Flowering period: period of flowering of plant species in Korea according to Lee (2003).

**Taxa**						**Resource**		**Flowering period[3)]**							
**Genus**	**species**	**Plant origin**	**Plant type**	**Corolla type**	**Color**	**Nectar**	**Pollen**	**Mar**	**Apr**	**May**	**Jun**	**Jul**	**Aug**	**Sep**	**Oct**
Actinidia	*arguta*	Native	W	Open	W	N	T			■	■				
	kolomikta	Native	W	Open	W	N	T				■	■			
	polygama	Native	W	Open	W	N	T				■	■			
Amorpha	*fruticosa*	North America	W	Tubular	Pu	G	G			■	■				
Aralia	*elata*	Native	W	Open	W	T	T					■	■	■	
Brassica	*napus*	Uncertain	H	Cruciform	Y	G	G		■						
	rapa var. *glabra*	Uncertain	H	Cruciform	Y	G	G	■	■	■					
	rapa var. *rapa*	Uncertain	H	Cruciform	W	G	G		■	■					
	oleracea var. *capitata*	Uncertain	H	Cruciform	Y	G	G			■	■				
	juncea	Uncertain	H	Cruciform	Y	G	G	■	■	■	■	■	■		
	juncea var. *crispifolia*	Uncertain	H	Cruciform	Y	G	G			■					
Capsicum	*annuum*	Central America	H	Open	W	T	T			■	■	■	■		
Castanea	*crenata*	Native	W	Open	W	E	E			■	■				
	mollissima	Native	W	Open	W	E	E			■	■				
Chelidonium	*majus* var. *asiaticum*	Native	H	Open	Y	I	I			■	■	■	■		
Corylus	*heterophylla*	Native	W	Open	R, G	N	E	■							
	sieboldiana	Native	W	Open	R, G	N	E	■							
	Sieboldiana var. *mandshurica*	Native	W	Open	R, G	N	E	■							
Diospyros	*lotus*	Native	W	Open	R, W	E	E				■				
Flueggea	*suffruticosa*	Native	W	Open	G, Y	T	T				■	■	■		

Mazus	*pumilus*	Native	H	Bilabiate	Pi, W	-	-
	miquelii	Native	H	Bilabiate	Pi, W	-	-
Oryza	*sativa*	Native	H	Open	G	N	G
Quercus	*acutissima*	Native	W	Open	G	N	T
	aliena	Native	W	Open	G	N	R
	variabilis	Native	W	Open	G	N	R
	serrata	Native	W	Open	G	N	R
	dentata	Native	W	Open	G	N	R
	mongolica	Native	W	Open	G	N	R
Rhus	*javanica*	Native, Oceania	W	Open	W	G	G
Toxicodendron	*succedaneum*	Native	W	Open	G	E	E
	sylvestre	Native	W	Open	G	E	E
	vernicifluum	India, China	W	Open	G	T	T
	trichocarpum	Native	W	Open	G	T	T
Ricinus	*communis*	Africa	H	Open	G	N	R
Robinia	*pseudoacacia*	North America	W	Papilionaceous	W	E	N
Rosa	*maximowicziana*	Native	W	Open	W	G	G
	wichuraiana	Native	W	Open	W	G	G
	multiflora	Native	W	Open	W	G	G
	rugosa	Native	W	Open	Pu	T	T
	acicularis	Europe, Asia, North America	W	Open	Pi	T	T
	davurica	Native	W	Open	Pi	T	T
Salvia	*plebeia*	Native, Oceania	H	Bilabiate	Pu	G	R
	splendens	South America	H	Bilabiate	R	T	T
	miltiorrhiza	China	H	Bilabiate	Pu	T	T
Zanthoxylum	*schinifolium*	Native	W	Open	G	R	R
